# The honeybee gut microbiome: a novel multidimensional model of antimicrobial resistance transmission and immune homeostasis from environmental interactions to health regulation

**DOI:** 10.1093/femsre/fuag001

**Published:** 2026-01-07

**Authors:** Qianmin Hai, Dufu Li, Tingyue Huang, Xiaoqun Dang, Jinshan Xu, Zhengang Ma, Zeyang Zhou

**Affiliations:** Key Laboratory of Pollinator Resources Conservation and Utilization of the Upper Yangtze River, Ministry of Agriculture and Rural Affairs, Chongqing Normal University, Chongqing 401331, China; Chongqing Key Laboratory of Vector Control and Utilization, College of Life Sciences, Chongqing Normal University, Chongqing 401331, China; Key Laboratory of Pollinator Resources Conservation and Utilization of the Upper Yangtze River, Ministry of Agriculture and Rural Affairs, Chongqing Normal University, Chongqing 401331, China; Chongqing Key Laboratory of Vector Control and Utilization, College of Life Sciences, Chongqing Normal University, Chongqing 401331, China; Key Laboratory of Pollinator Resources Conservation and Utilization of the Upper Yangtze River, Ministry of Agriculture and Rural Affairs, Chongqing Normal University, Chongqing 401331, China; Chongqing Key Laboratory of Vector Control and Utilization, College of Life Sciences, Chongqing Normal University, Chongqing 401331, China; Key Laboratory of Pollinator Resources Conservation and Utilization of the Upper Yangtze River, Ministry of Agriculture and Rural Affairs, Chongqing Normal University, Chongqing 401331, China; Chongqing Key Laboratory of Vector Control and Utilization, College of Life Sciences, Chongqing Normal University, Chongqing 401331, China; Key Laboratory of Pollinator Resources Conservation and Utilization of the Upper Yangtze River, Ministry of Agriculture and Rural Affairs, Chongqing Normal University, Chongqing 401331, China; Chongqing Key Laboratory of Vector Control and Utilization, College of Life Sciences, Chongqing Normal University, Chongqing 401331, China; Key Laboratory of Pollinator Resources Conservation and Utilization of the Upper Yangtze River, Ministry of Agriculture and Rural Affairs, Chongqing Normal University, Chongqing 401331, China; Chongqing Key Laboratory of Vector Control and Utilization, College of Life Sciences, Chongqing Normal University, Chongqing 401331, China; Key Laboratory of Pollinator Resources Conservation and Utilization of the Upper Yangtze River, Ministry of Agriculture and Rural Affairs, Chongqing Normal University, Chongqing 401331, China; Chongqing Key Laboratory of Vector Control and Utilization, College of Life Sciences, Chongqing Normal University, Chongqing 401331, China

**Keywords:** antimicrobial resistance transmission, honeybee gut microbiome, horizontal gene transfer, host–microbe interactions, bee model, One Health

## Abstract

The honeybee gut microbiome has emerged as a model system in microbial ecology, valued for its structural stability and host specificity, and has garnered significant attention for elucidating universal principles of host–microbe interactions. This review advocates for the honeybee as a multidisciplinary model organism, highlighting the unique role of its gut microbiota in maintaining colony immune homeostasis, driving host co-evolution, unraveling the transmission mechanisms of antibiotic resistance genes (ARGs), and enhancing host adaptability to environmental stressors. By integrating multidimensional factors, including environmental gradients and apicultural practices, we construct an “Environment–Microbiota–Host Health” interaction framework to transcend the limitations of single-factor analyses. This framework provides a novel paradigm for the ecological containment of antimicrobial resistance, the conservation of pollinator resources, and microbiome-based engineering interventions. The review underscores the unique value of the honeybee model in unraveling social insect-microbe coevolution and resistance transmission dynamics, while also prospecting its application potential in developing novel antimicrobial peptides, designing probiotic formulations, and monitoring environmental resistance.

## Teaser

This review establishes the honey bee gut microbiome as an integrative model system, synthesising its pivotal role in antimicrobial resistance dissemination through specialised plasmid evolution, strain-level immune specificity, and environmental adaptation, thereby bridging fundamental microbial ecology with actionable One Health solutions.

Abbreviation list AMPs: Antibacterial Peptides
ARGs: Antibiotic Resistance Genes
ARTP: Atmospheric and Room Temperature Plasma
AMR: Antimicrobial Resistance
ARB: Antibiotic Resistant Bacteria
ASOs: Antisense Oligonucleotides
ARGs-GIS: Antibiotic Resistance Genes-Geographic Information System
BGCs: Biosynthetic Gene Clusters
CCD: Colony Collapse Disorder
CNS: Central Nervous System
CRISPR: Clustered Regularly Interspaced Short Palindromic Repeats
DWV: Deformed Wing Virus
DUOX: Dual Oxidase
HGT: Horizontal Gene Transfer
IncQ: *Escherichia coli* Incompatibility Group Q
MAPK: Mitogen-Activated Protein Kinase
MGBA: Microbiome-Gut-Brain Axis
MGEs: Mobile Genetic Elements
PG: Prostaglandin
PGRP-LB: Peptidoglycan Recognition Protein LB
PGRP-LC: Peptidoglycan Recognition Protein LC
PGRP-SC: Peptidoglycan Recognition Protein SC
PRRs: Pattern Recognition Receptors
RTX: Repeats in Toxin
RNAi: RNA interference
RiPPs: Post-translationally Modified Peptides
ROS: Reactive Oxygen Species
SynComs: Synthetic microbial Communities

## Introduction

The gut microbiota is crucial in regulating host development and physiology, including metabolism and immune function. Commensal microorganisms have a significant influence on the central nervous system and behavioral processes in humans and several animal models (Zhang et al. 2022a). Microbes can modulate host brain function through various pathways, such as immune regulation, and via microbial metabolites involved in the gut–brain axis (Cryan and Dinan 2012). Moreover, the microbiome is essential for numerous biochemical and physiological processes and can markedly affect host health, immune modulation, growth, development, and even behavior through this axis (Rowland et al. 2018, Evariste et al. 2019, Hou et al. 2022). However, most current research on gut microbes focuses on mammalian and non-social insect models. Elucidating the role of individual gut members in mammals remains challenging, partly due to the complexity and unpredictability of gut communities, as well as difficulties in maintaining and manipulating germ-free animals (Pfeiffer and Virgin 2016). In this context, insects have emerged as powerful and tractable model systems to decipher host–microbiota interactions. In contrast to the high diversity typical of mammalian guts, many insects harbor simplified and more specialised microbial communities, offering a reductionist advantage for mechanistic studies (Engel and Moran 2013). Particularly, social insects such as bees, ants, and termites represent exceptional models. Their intricate social behaviors facilitate reliable microbial transmission among colony members, fostering the evolution of stable, functionally specialised gut consortia that are integral to host nutrition, protection, and social physiology (Liberti et al. 2024). Therefore, a model exhibiting high sociality and a specialised gut community would be ideal for comprehensively understanding the relationship between gut microbiota and host social behavior. The gut microbiota influences many aspects of insect life, including food digestion, nutrient production, pathogen protection, survival, and reproduction—all of which significantly shape insect life history (Daisley et al. 2020b, Wang et al. 2019, Allara and Girard 2024). Various insects rely on symbiotic bacteria for essential functions: in *Drosophila melanogaster, Acetobacter* and *Lactobacillus* species regulate growth and immunity and accelerate larval development (Engel and Moran 2013, Shuttleworth et al. 2019); in *Ceratitis capitata, Klebsiella oxytoca* and *Enterobacter* spp. promote larval growth through nutrient synthesis and antibacterial compound production, playing a key role in sterile insect technique programs (Augustinos et al. 2015); and in *Plutella xylostella*, germ-free larvae exhibit reduced survival and pupal weight, which can be significantly restored by inoculation with *Enterobacter cloacae* (Somerville et al. 2019). Furthermore, due to their unique viviparous reproductive mode, *Glossina* spp. transmit endosymbionts such as *Wigglesworthia* and *Sodalis* to intrauterine larvae through maternal transmission. *Wigglesworthia* is vertically transmitted through the mother and can regulate the intestinal immune protein (Peptidoglycan recognition protein LB, PGRP-LB) of tsetse flies and affect the formation of peritrophic matrix, thereby inhibiting trypanosome infection; while *Sodalis*, also a maternally transmitted symbiont, has a density positively correlated with the trypanosome infection rate and may promote infection (Weiss et al. 2019). In the *Bombyx mor* and the *Galleria mellonell, Lactobacillus* strains exhibit antimicrobial activity against pathogens such as *Pseudomonas aeruginosa, Escherichia coli*, and *Candida albicans* (Nishida et al. 2017, Ribeiro et al. 2017, Jorjao et al. 2018). This suggests that the probiotic function of these lactobacilli may stem not only from direct antibacterial effects but also from the activation or synergy with the host’s innate immune system, thereby enhancing overall anti-infection capacity.

**Figure 1:**
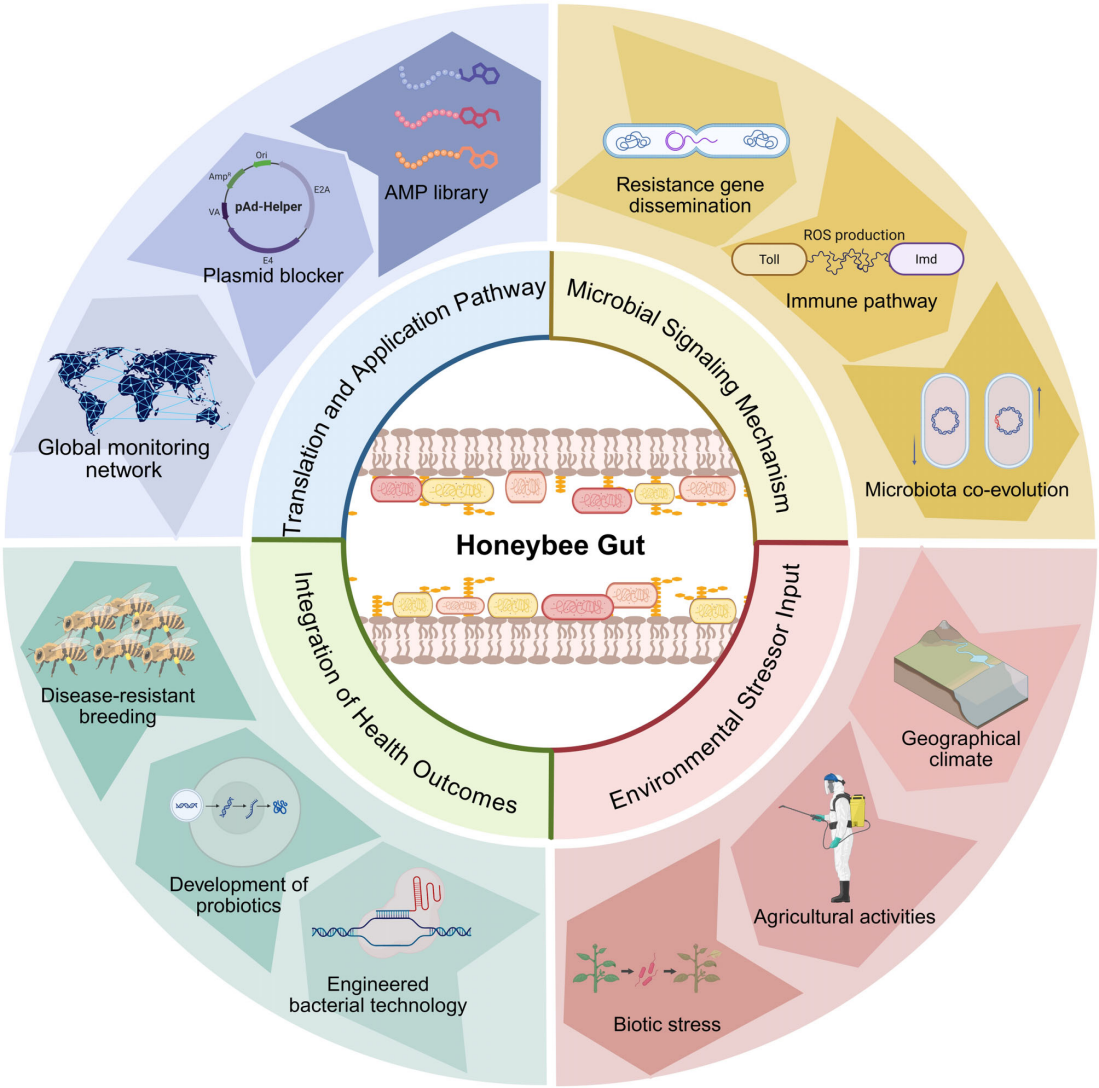
Multidimensional interaction framework and application prospects of bee gut microbiota. The honeybee gut as a dynamic interface at the nexus of environmental pressures and host health. Central to this model is the core gut microbiota, whose composition and social transmission drive specific response mechanisms to environmental stressors. These microbial functions directly influence host health outcomes, while also enabling transformative applications for sustainability and disease management, thereby closing the loop from fundamental ecology to One Health solutions. Created with BioRender.

The 2025 report of the World Health Organization (WHO) pointed out that in 2023, one-sixth of the world’s common bacterial infections had been resistant to antibiotics, and the resistance of more than 40% of the monitored antibiotics from 2018 to 2023 was still rising at an average annual rate of 5%–15% (World Health Organization, 2025). As the key guardian of global food security and biodiversity, the pollinator population represented by bees was declining sharply (Winfree et al. 2009). The honey bee gut microbiome serves as a dynamic, interactive interface between the host and its environment, playing a central role not only in maintaining colony health but also in the dissemination of antibiotic resistance genes (ARGs), regulating immune homeostasis, and facilitating environmental adaptation. Characterised by a highly simplified community structure (5–9 core bacterial genera) and efficient social transmission networks, the bee gut ecosystem offers a unique model for deciphering the mechanisms of resistance spread, immune regulation, and host adaptation (Sun et al. 2022). Against the backdrop of global antimicrobial resistance (AMR) escalation and pollinator decline, research on this system has progressed beyond mere microbial profiling toward an integrated “Environment-Microbiota-Host” multidimensional framework. The *Apis cerana*, with its fixed foraging radius (approximately 3 km) and semi-wild management, exhibits an intestinal resistome that accurately reflects regional pollution levels—ARG abundance in colonies from the North China Plain is significantly higher than that in the Qinghai–Tibet Plateau, correlating strongly with veterinary antibiotic usage and underscoring its value as a bio-sentinel for environmental resistance monitoring (Sun et al. 2022). Resistance genes (e.g. *strA, sul2*) can be efficiently transferred among gut bacteria via *E. coli* incompatibility group Q (IncQ plasmids) featuring a “satellite structure” (lacking *mobABC*/*repABC* regions), leading to enhanced conjugation efficiency (Zhang et al. 2019). Through trophallaxis among worker bees, these ARGs disseminate throughout the colony within 72 h, ultimately establishing the queen as a reservoir of resistance genes (Wang et al. 2024), thereby accelerating ecological risk propagation. Furthermore, the gut microbiota engages in refined “metabolism-immunity” collaboration to counteract environmental stressors. For instance, *Gilliamella* can activate the gut–brain axis to mitigate pesticide-induced damage and improve colony survival (Zhao et al. 2025). *Bombella* modulates tryptophan metabolism to extend worker lifespan (Jin et al. 2025). We propose a novel multi-dimensional interaction paradigm that systematically integrates resistance transmission networks, host–microbe coevolution mechanisms, and environmental response strategies, thereby offering a scientific engine for curbing ARG dissemination, optimising colony health, and advancing One Health practices. Consequently, the honeybee transcends the role of a conventional model organism for a single discipline. It emerges as a multidisciplinary model organism—a tractable biological system that uniquely integrates core questions from ecology, microbiology, immunology, and public health within a unified “Environment–Microbiota–Host” framework (Fig. 1). This integrative capacity allows researchers to bridge methodologies from molecular genetics to field ecology, and to derive generalisable insights into complex cross-species issues, such as the ecological dissemination of AMR. The following sections articulate this multidimensional model, detailing its fundamental characteristics (Section 1), co-evolutionary (Section 2) and transmission mechanisms (Section 3), its responses to environmental gradients (Section 4), and its translational potential (Section 5).

## The honeybee gut microbiome: from fundamental characteristics to emergence as a model system

This section systematically elaborates the highly simplified and conserved core features of the honeybee gut microbiota, establishing its theoretical and experimental advantages as an ideal model for studying host–microbe interactions.

### Impact of gut microbiota on host physiology

Beyond their critical role as pollinators, honeybees possess a distinctive gut microbiota that is integral to their physiology and sociality (Callegari et al. 2021). This community, known for its stability and conserved functions in metabolism and defense, offers a unique biological template for research (Kwong and Moran 2016, Zheng et al. 2018). Therefore, in-depth research into the dynamics of the honeybee microbiota not only offers novel insights into host–microbe interactions, but also contributes to the improvement of honeybee health and the development of apiculture (Hroncova et al. 2019). Compared with the complex gut microbiota of mammals, the gut communities of both *A. cerana* and *Apis mellifera* honeybees are relatively simple and host-specific, dominated by only 5–9 core bacterial genera—including *Gilliamella, Snodgrassella, Bartonella, Bifidobacterium*, and *Lactobacillus Firm-4* and *Firm-5* clades—which collectively account for over 95% of the total gut bacteria (Kwong and Moran 2016). Among these core taxa, *Gilliamella* degrades pollen pectin to enhance nutrient utilisation and produces antibacterial peptides (AMPs) that help combat pathogens (Lang et al. 2023a, Engel et al. 2012). Moreover, colonisation by *Gilliamella* significantly promotes lipid synthesis and thermogenesis in bees, reflecting metabolic synergy between the host and its symbionts (Tang et al. 2025). *Snodgrassella alvi* contributes to host physiology and health maintenance, in part by regulating the expression of AMPs (Kwong et al. 2017, Horak et al. 2020), while *Lactobacillus* species aid in digesting complex carbohydrates and produce antimicrobial compounds that inhibit pathogen colonisation (Vasquez et al. 2012). This conserved microbiota makes the honey bee a tractable, realistic, and constrained ecosystem for studying the transfer and maintenance of ARGs among gut bacteria (Zheng et al. 2018). The low complexity of this system offers an ideal platform for precisely dissecting microbial functions and interaction mechanisms. Notably, all core honey bee gut bacteria have been successfully isolated and cultured *in vitro*, and most strains are amenable to genetic manipulation, greatly facilitating related molecular mechanistic studies (Sun et al. 2022).

### The honeybee as a model system for animal gut microbiota research: a comparative perspective

Honey bees can be raised germ-free and have a short gut colonisation cycle (approximately 3–5 days), therefore exhibiting high experimental feasibility (Kwong et al. 2014b, Hroncova et al. 2015). Typically, the core microbiota can complete colonisation and establish a stable community structure by Day 4 (Powell et al. 2014, Ellegaard and Engel 2019). During this critical window, transient immune suppression facilitates the stable establishment of symbionts (Guo et al. 2023). Meanwhile, the successful establishment of an *in vivo* model for monitoring the horizontal transfer of ARGs enables the direct, real-time observation of plasmid transmission among gut bacteria (Sun et al. 2022). Honeybees also serve as ecological indicators. Their fixed foraging behavior (Ludvigsen et al. 2018) makes them effective “live samplers” of environmental microbes and pollutants. Specifically, the *A. cerana*, with its semi-wild rearing habits and geographical sedentariness, undergoes minimal artificial domestication. This enables it to sensitively reflect regional differences in antibiotic selection pressure (Ji et al. 2020). Consequently, the composition of the gut resistome in these bees can be used as a biomarker for monitoring environmental levels of AMR. Furthermore, as eusocial insects, trophallaxis among worker bees constitutes a microbial social transmission network (Martinson et al. 2012, Powell et al. 2014), offering a unique model for investigating microbial spread and evolution at the group level (Table 1). This model is significant for exploring the transmission dynamics of ARGs within colonies. The innovative potential of this model can steer gut microbiome research from a “single-microbiota functional analysis” paradigm toward an “environment–host–microbiota multidimensional interaction” framework, laying a methodological foundation for subsequent studies on the dissemination of resistance and immune homeostasis. A systematic comparison between the honeybee model and traditional mammalian models not only highlights its distinctive advantages but also clarifies its irreplaceability in addressing specific scientific questions. In contrast to the extreme complexity and high inter-individual variability of mammalian gut microbiota, the honeybee gut community is simplified, stable, and highly host-specific (Kwong and Moran 2016). This low complexity provides an ideal basis for precisely dissecting microbial functions and interaction mechanisms. Moreover, the high genetic uniformity among worker bees within a colony, combined with their natural social transmission network maintained through trophallaxis, makes honeybees a special model for studying the spread dynamics of both microbiota and ARGs at the population level (Wang et al. 2024). Notably, the role of epigenetic mechanisms (e.g. DNA methylation) in mediating the long-term effects of environmental stress on host–microbiota interactions has become a cutting-edge focus in mammalian research. (Pepke et al. 2024). Introducing such research into the honeybee model could help build a bridge between insect and mammalian systems, revealing conserved regulatory mechanisms across species.

**Table 1:** Comparison of the bee gut microbiome with traditional gut microbiota model systems

Feature	Bee model	Rodent model	*In vitro* culture system	References
Core microbiota complexity	Low (5–9 core genera)	High (>200 genera)	Adjustable but with low ecological relevance. Unable to simulate the complex physiological environment and microbial interactions within the host’s body.	(Yang et al. 2025)
Aseptic model construction cycle	Short (<7 days)	Long (>21 days)	Not applicable	(Sun et al. 2022)
Social Communication Simulation Ability	Natural support group dissemination	Manual intervention is required	Cannot be achieved	(Mee and Barribeau 2023)
Environmental indication sensitivity	Height (reflecting a 3-km radius environment)	Low (basically not used for environmental indication research)	None	(Sun et al. 2022)
Advantages of drug resistance research	High ecological relevance; can directly study the microbial response and resistance transmission under agricultural antibiotic/pesticide exposure; it has been confirmed that ARGs can be transmitted through plasmid-mediated horizontal gene transfer.	Predicting human reactions is better, but the ecological authenticity is lower.	Mainly used for initial mechanism exploration and high-throughput screening, it cannot reflect overall physiology.	(Sun et al. 2022)
Operation and genetic background	The genetic background is highly consistent, with small individual differences within the bee colony and good experimental reproducibility	There are genetic and physiological differences between individuals, and inbreeding is needed to reduce bias.	It can precisely control conditions, with the highest repeatability, but detached from the internal environment.	(Tang et al. 2025)
Research on manipulability and genetic tools	Genetic tools are becoming increasingly sophisticated; engineering bacteria can be used for genetic manipulation; preliminary establishment of a bacteriophage-bacteria interaction research model.	Genetic manipulation technology is mature but has a long cycle and high cost; it can be used for complex gene editing.	The operation is the simplest and is suitable for large-scale gene function screening.	(Ndiaye et al. 2025) (Tang et al. 2025)
Typical Applications and Core Discoveries	1. Reveal that the probiotic *Gilliamella* sp. G0441 enhances pesticide tolerance through the gut–brain axis. 2. Draw a national map of bee gut resistance genes and reveal the transmission mechanism of ARGs. 3. Reveal that bees and *Gilliamella* enhance the host’s ability to keep warm through synergistic sugar metabolism. 4. Explain how the closely related *Gilliamella* strain achieves coexistence through niche differentiation and differences in life history strategies.	Evaluate the safety, efficacy, and pharmacokinetics of drugs in complex physiological systems.	Used for basic biological research such as drug toxicity screening and cell signaling pathways。	(Sun et al. 2022) (Zhao et al. 2025) Tang et al. 2025) (Yang et al. 2025)
Depth of Host Microbial Interaction Research	Can clearly analyze the complete pathway from microbial function to host phenotype.	Suitable for complex systemic physiological research, but mechanism analysis is more challenging due to the complexity of the system.	Limited to the study of microscopic mechanisms at the cellular or molecular level.	(Zhao et al. 2025)
Ethical restrictions	Lower restrictions and less controversy. As invertebrates, they are usually less subject to animal welfare regulations. Its research is often regarded as conforming to the 3R principle of “replacement” and “reduction”, which has caused the least ethical controversy. Experiments are usually conducted in natural social groups with fewer behavioral restrictions.	Strict restrictions and complex review. As a mammal, it is regulated by strict laws and regulations and the institutional animal care and use committee. All experimental protocols need to be reviewed in detail by the ethics committee in advance to ensure that the necessity is fully demonstrated, the number of animals used is minimised, and the experimental procedures are optimised to reduce pain. Research involving high pain or mental pain faces higher approval thresholds and public ethical concerns.	None	(Meerburg et al. 2008)
Cost input	The cost is significantly low. The colony can maintain and reproduce by itself, and the initial acquisition and long-term feeding costs are low. No expensive temperature control, aseptic isolation, or large cage facilities are required. The cost of daily consumables is very low. Behavioral experiments are simple.	High cost. It needs to be purchased from commercial breeding institutions, and the price of the strain animals is not low. The requirements for a barrier environment are strict, and the cost of facility construction and maintenance is extremely high. Cages, bedding, special feed and veterinary care constitute ongoing daily expenses.	Lower	(Cini et al. 2025)

## Host–microbe coevolution: immune recognition and homeostasis maintenance mechanisms

This part deciphers the sophisticated immune mechanisms, such as the “dual-pathway recognition–precision reactive oxygen species clearance” strategy, that enable honeybees to distinguish commensals from pathogens, revealing evolved regulatory strategies for maintaining host–microbe homeostasis.

### Immune recognition in host–microbe coevolution

**Figure 2:**
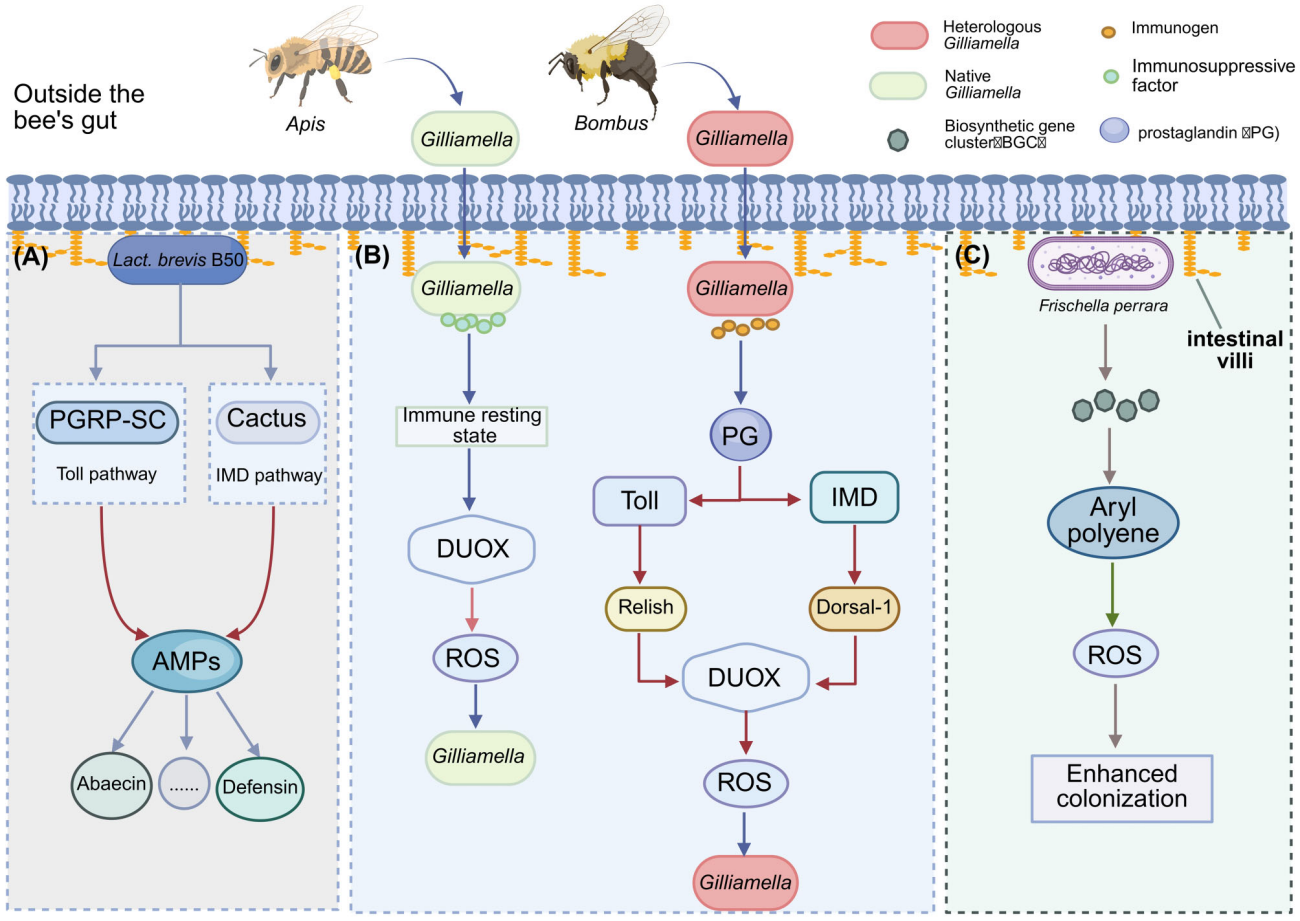
The precise recognition mechanism in honeybee gut immunity. (A) *Lactobacillus brevis* B50 promotes the release of AMPs by regulating the Toll and IMD pathways. (B) Heterologous *Gilliamella* regulates the production of ROS through the DUOX-ROS system, determining colonisation status. (C) BGC produced by *F. perrara* can resist the ROS produced by the host, allowing microbial colonisation. AMPs: antibacterial peptides; DUOX: dual oxidase; PG: prostaglandin; ROS: reactive oxygen species; PGRP-SC: Peptidoglycan recognition protein SC. Created with BioRender.

During long-term co-evolution with gut microbes, honeybees have developed a sophisticated immune recognition system capable of distinguishing commensal strains from pathogens, and even identifying subtle differences among closely related bacterial species (Fig. 2B). This system enables precise defense against pathogens and regulation of the gut microbiota (Salzman et al. 2010, Yao et al. 2016, Marra et al. 2021). In the insect gut, reactive oxygen species (ROS)—primarily generated by the dual oxidase (DUOX) system—constitute a nonspecific bactericidal mechanism that typically serves as the first line of defense against pathogens (Bai et al. 2021). In honeybees, the DUOX system expressed in intestinal epithelial cells is activated with high selectivity: it responds to alien bacterial strains while maintaining immune tolerance toward native symbionts (Guo et al. 2023). Furthermore, host–gut bacteria specificity has been confirmed between *Apis* and *Bombu*, where native strains of core gut bacteria such as *S. alvi* and *Lactobacillus Firm-5* show higher colonisation success in their original hosts (Kwong et al. 2014a, Ellegaard and Engel 2019). Studies indicate that *Lactobacillus brevis* B50 enhances gut immunity by promoting antimicrobial peptide (Abaecin, Defensin) expression and modulating key genes of the Toll and Imd pathways (Dorsal, Cactus, Kenny, Relish) (Maruscakova et al. 2020). This immunomodulation involves specific interactions: surface proteins of *L. brevis* engage with Peptidoglycan recognition protein LC (PGRP-LC) in the Toll pathway and Cactus in the Imd pathway, thereby activating the innate immune system in bumblebees (Lang et al. 2022). Further evidence suggests additional binding to Cactus (IMD pathway) and Peptidoglycan recognition protein SC (PGRP-SC) (Toll pathway), which synergistically upregulate AMP expression to bolster immune competence (Yu et al. 2024) (Fig. 2A). Secondary metabolites are small molecules typically produced by enzymes encoded in biosynthetic gene clusters (BGCs). Recent studies have identified potential BGCs in bee gut bacteria such as *Apilactobacillus kunkeei* (Zendo et al. 2020) and *Frischella perrara* (Schmidt et al. 2023). The resulting secondary metabolites are believed to mediate microbe-microbe and microbe-host interactions. For instance, *F. perrara* carries a BGC for aryl polyene biosynthesis, which may facilitate its persistent colonisation in the hindgut by conferring resistance to host-derived ROS (Schmidt et al. 2023) (Fig. 2C). Currently, genomic mining strategies that identify BGCs in bacterial genomes and predict the chemical structures of their products have revealed numerous such clusters in host-associated symbionts (Kissoyan et al. 2019, Anderson and Fernando 2021, Zhang et al. 2023). Subsequent systematic and in-depth exploration is likely to uncover more novel natural products derived from the honeybee gut microbiota.

Moreover, when the honeybee gut is colonised by *Gilliamella* strains derived from bumblebees, prostaglandin (PG) acts as a key signaling molecule that significantly enhances both the Toll and IMD immune signaling pathways, thereby triggering a DUOX-ROS cascade that ultimately clears these foreign bacterial strains. In contrast, native honeybee-derived *Gilliamella* strains evade this immune activation through the secretion of specific immunosuppressive factors. Within this mechanism, the transcription factors Relish and Dorsal-1 likely contribute to ROS generation by modulating the expression of DUOX. Thus, by driving such differential immune responses, the honeybee host successfully establishes an intestinal microenvironment that excludes foreign bacteria while preserving colonisation by its native symbionts, thereby ensuring symbiotic specificity. This represents a highly efficient evolutionary adaptation of pre-existing anti-pathogen mechanisms (Guo et al. 2023). The “dual-pathway recognition–precision ROS clearance” model illustrates the strain-level discriminative capacity of the insect immune system, offering a molecular perspective on host-symbiont coevolution. Although this model elucidates the downstream execution of immune specificity, the initial host sensors responsible for distinguishing between conspecific bacterial strains remain enigmatic. Identifying the precise pattern recognition receptors (PRRs; Buchon et al. 2014) that detect strain-specific surface motifs constitutes a fundamental gap in our understanding and represents a major goal for future research. While the “dual-pathway recognition–precision ROS clearance” model elucidates the downstream execution of immune specificity, the initial host sensors responsible for distinguishing between congeneric bacterial strains remain enigmatic. The identity of the precise PRRs (Buchon et al. 2014) that detect strain-specific surface motifs constitutes a fundamental gap in our understanding, representing a prime target for future research.

### Microbial functionality and host coevolutionary selection

**Figure 3:**
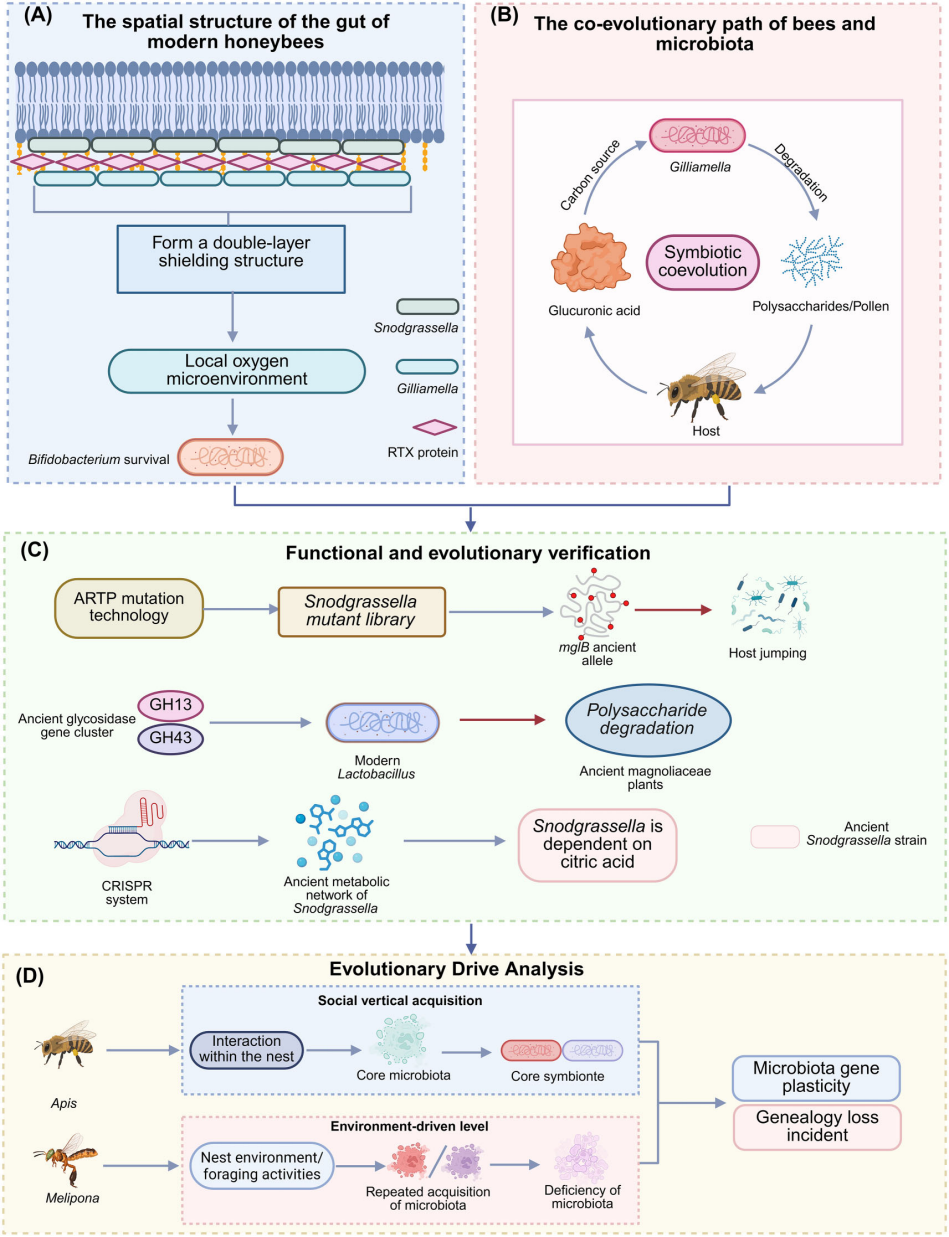
Spatial colonisation patterns of bee gut symbiotic bacteria and host–microbe co-evolution mechanism. (A) The spatial structure of the gut of the modern honeybee. The double-layered shielding structure formed by modern honeybees can better maintain the survival of obligate anaerobic bacteria. (B) The co-evolutionary path of bees and microbiota. *Gilliamella* and bees adjust their metabolism under cold conditions, thereby promoting co-evolution. (C) Functional and evolutionary verification. Verification of the differences in microbial functions between ancient and modern bee intestines, and the emergence of co-evolution, through techniques such as gene reconstruction by ARTP and CRISPR. (D) Evolution-driven analysis. Above: In *Apis*, core symbionts are stably inherited through social transmission within the hive. Below: In stingless bees (*Melipona*), microbiota are primarily reacquired from the environment each generation, leading to a variable community lacking those core symbionts but with higher genomic plasticity. This illustrates a key evolutionary divergence in social insect-microbe symbiosis. Created with BioRender.

The selectivity of honeybees toward their gut microbiota is manifested not only at the immune level but also in the precise regulation of the microbial spatial architecture (Fig. 3A). Core symbiotic bacteria could form specialised biofilm structures within the honeybee gut: *Snodgrassella* colonises the epithelium of the anterior ileum, forming a continuous biofilm layer, while *Gilliamella* embeds within its outer region, collectively constituting a “dual-layer shield.” This structure not only reinforces the host defense barrier and elicits immune responses but also helps maintain the survival of obligate anaerobes (e.g. *Bifidobacterium*) by modulating the local oxygen microenvironment (Horak et al. 2020). Beyond the physical barrier, defensive functions can also be mediated by identified secretion systems or repeats in toxin (RTX) proteins. In particular, RTX proteins may act as bacteriocins or contribute to defense against environmental stressors by forming protective surface layers or facilitating intercellular adhesion within biofilms, thereby serving as key determinants of such defensive functions (Satchell 2011). Additionally, RTX proteins may play a role in establishing the close association between *Snodgrassella* and gut epithelial cells after pupal eclosion, thereby influencing the development of the newly formed intestine in adult bees (Martinson et al. 2012). Studies on Eastern honeybees across different altitudes in the Indian Peninsula have revealed that genomic plasticity in *Gilliamella* enables dynamic adjustment of polysaccharide metabolic gene expression, allowing adaptation to variations in nectar sources along altitudinal gradients (Kwong and Moran 2013). This reflects functional adaptation and co-evolution between the gut microbiota and the host ecological niche.

The evolutionary trajectory of honeybee-gut microbiota symbiosis is evidenced by sophisticated molecular adaptations shaped through long-term coevolution. Recent research demonstrates that the core gut bacterium *Gilliamella* has evolved a specialised metabolic partnership with its honeybee host to enhance cold adaptation. In cold-tolerant bees, *Gilliamella* acts as an “energy assistant,” efficiently degrading pollen polysaccharides into glucose and pyruvate—key substrates for host thermogenesis—while limiting its own consumption of these products. In return, the host bee upregulates the synthesis of glucuronic acid as a preferred carbon source for the bacterium, forming a reciprocal “metabolic contract” that underscores a finely tuned, co-adaptive relationship (Tang et al. 2025). Furthermore, comparative metagenomic studies across diverse honeybee species reveal that evolutionary processes such as gene gain and loss, along with the adaptation of core bacterial lineages to specific host species, have been fundamental in shaping the functional landscape of the gut microbiome (Prasad et al. 2025). These insights illustrate how both metabolic integration and genomic evolution serve as key drivers in the ongoing coevolution between honeybees and their gut microbiota (Fig. 3B). In contrast to honeybees, which primarily acquire and stably inherit their core gut microbiota through social interactions within the hive, *Melipona* exhibit a more open, environmentally driven acquisition mode for their microbial symbionts. Their gut communities commonly lack the core symbiotic bacteria *Snodgrassella* and *Gilliamella* found in honeybees, instead encompassing a greater proportion of bacteria and yeasts that are horizontally acquired from the environment. Consequently, *Melipona* likely reacquires its symbionts anew each generation primarily from the nest environment and foraging activities. This mode fosters higher genomic plasticity within its symbiotic microbiota and enables the adaptive integration of novel environmental strains into obligate symbiotic partnerships (Cerqueira et al. 2024). Comparative analysis of microbiota between *Apis* and *Melipona* revealed the absence of *Snodgrassella* and *Gilliamella* in the latter, illustrating a lineage-specific loss event during the early divergence of eusocial bees (Cerqueira et al. 2021) (Fig. 3D). A *Snodgrassella* mutant library constructed using Atmospheric and Room Temperature Plasma (ARTP) mutagenesis showed that introducing the ancient *mglB* allele significantly enhanced colonisation in non-native hosts, confirming that paleoalleles facilitate host jumping by regulating type IV pilus motility (Meng et al. 2024). Gnotobiotic bees colonised with reconstructed ancestral microbiota triggered a DUOX-ROS immune response only half as strong as that induced by modern microbiota, indicating host immune pressure as a key evolutionary force driving strain-specific adaptation (Guo et al. 2023). Based on a paleopan-genomic database of amber-derived *Gilliamella*, a synthetic biology team reconstructed ancestral glycoside hydrolase gene clusters (GH13,GH14) and heterologously expressed them in modern *Lactobacillus*, demonstrating higher degradation efficiency for ancestral *Magnoliophyta polysaccharides* than modern enzymes (Cerqueira et al. 2024), supporting the Cretaceous bee-angiosperm co-radiation hypothesis (Peris and Condamine 2024). Using a Clustered Regularly Interspaced Short Palindromic Repeats (CRISPR) activation system to reconstruct ancient metabolic networks in *Snodgrassella*, researchers found reliance on host-derived citrate rather than the glycerate utilised by modern strains, reflecting an evolutionary shift in the gut microenvironment from acidic to weakly alkaline conditions (Quinn et al. 2024) (Fig. 3C).

Host selection of gut microbiota is stringent and evolutionarily honed. This selective pressure is exerted and maintained through the colony’s social fabric. Frequent social interactions, primarily trophallaxis and exposure to nestmates’ feces, serve as the crucial transmission network through which newly emerged workers acquire their core microbiota. However, this same network also functions as a filter and stabiliser: it consistently propagates host-adapted symbionts while limiting the establishment of non-native strains, thereby ensuring microbial stability across generations. This mechanism elucidates the stable inheritance of gut microbiota in social insect colonies and identifies the early adult stage as a key temporal window for manipulating microbiota to improve colony health.

## Transmission mechanisms and ecological risks of AMR in the honeybee gut microbiome

This chapter focuses on plasmid-mediated horizontal gene transfer, clarifying the ecological role of the honeybee gut as a hub for ARG dissemination and evaluating its potential risks through social behavioral transmission within colonies.

### Dissecting the transmission mechanisms of antibiotic resistance in the honeybee gut

**Figure 4:**
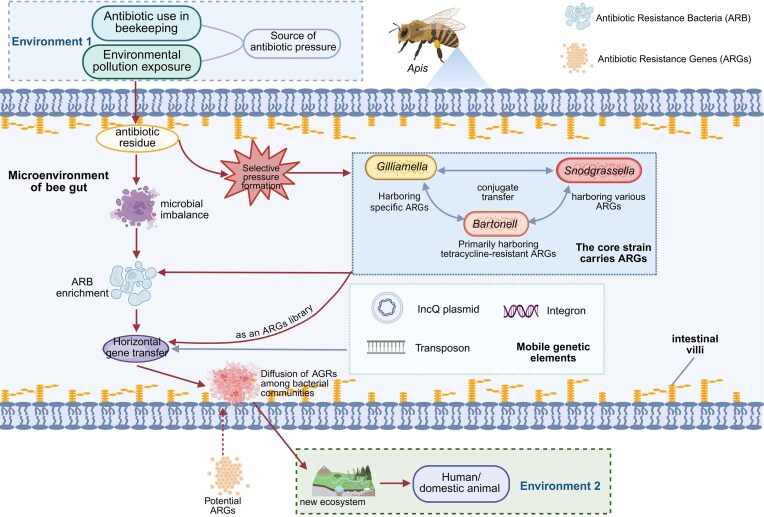
Transmission and diffusion mechanism of ARGs in the bee gut. ARGs can enter the bee gut from environment 1, and different ARGs can undergo conjugate transfer by binding to mobile elements in the core microbiota of the bee gut. Eventually, the bees introduce ARGs into environment 2, causing their diffusion. Meanwhile, potential ARGs from other environments can also directly enter the gut microbiota of bees, allowing them to enter the transmission pathway. Created with BioRender.

The global AMR crisis demands innovative model systems to unravel the complex interactions between environmental pressures, microbial communities, and host responses. The honey bee gut microbiome, with its remarkable simplicity and social transmission dynamics, has emerged as such a system—providing insights that extend far beyond pollinator health to fundamental ecological and biomedical principles. The spread of antibiotic resistance constitutes a global public health crisis, and the honeybee gut microecosystem has become a covert arena for ARG dissemination (Fig. 4). Antibiotic residues not only negatively impact bee product quality but also drive the emergence of resistant pathogens (Evans 2003) and disrupt the natural gut microbial community. Under antibiotic selective pressure, resistant bacteria (ARB) proliferate and facilitate the horizontal transfer of ARGs among gut bacteria (Ludvigsen et al. 2018). Such transfer may further enable ARG dissemination to new pollination sites during foraging activities (McManus et al. 2002). Moreover, *tetR* genes are consistently associated with mobile genetic elements showing high similarity to those identified in human pathogens and livestock, suggesting that bees may act as intermediaries in the environmental spread of clinically relevant ARGs (Alippi et al. 2014). Metagenomic analyses have revealed 27 classes of resistance genes in the Chinese honeybee gut microbiome, covering clinically common antibiotics such as β-lactams, tetracyclines, and sulfonamides, with *strA* and *sul2* genes being particularly prevalent. Comparative studies show that Western honeybees (under migratory beekeeping) harbor significantly higher ARG abundance than native Asian honeybees (under semi-wild management), indicating that human activities—such as migratory beekeeping and hive disinfection—exacerbate environmental antibiotic exposure and directly promote ARG enrichment in the gut (Sun et al. 2022). Mobilisable plasmids mediate conjugative transfer of ARGs among core gut bacteria, including *Gilliamella, Snodgrassella*, and *Bartonella*, which are gut-specific symbionts, illustrating horizontal gene transfer as a key mechanism for ARG dissemination within the gut community. Most ARGs are carried by *Gilliamella*, while *Snodgrassella* serves as the primary reservoir for tetracycline resistance genes (Sun et al. 2022). Previous screening of honeybee guts also indicated that the majority of tetracycline-resistant clones belong to *Snodgrassella*, which frequently carries tetracycline resistance determinants (Tian et al. 2012). The association of ARGs with mobile genetic elements such as plasmids and transposons is essential for facilitating the spread of resistance across environmental boundaries (Bengtsson-Palme et al. 2018). Bacteria facilitate the dissemination of ARGs via horizontal gene transfer (HGT), particularly when ARGs are harbored on mobile genetic elements (MGEs) such as transposons and conjugative elements (Inda-Diaz et al. 2023). The mechanisms underpinning bacterial antibiotic resistance are multifaceted, encompassing the production of inactivating enzymes (e.g. β-lactamases capable of hydrolyzing penicillins or cephalosporins), alterations in membrane permeability, and structural modifications of bacterial target sites (Abbas et al. 2024), all collectively contributing to antibiotic treatment failure. Antibiotic exposure during the larval stage induces sustained physiological impacts: recent evidence indicates that larval contact with oxytetracycline persistently affects adult bee weight, gene expression profiles, and metabolic processes, while concurrently compromising the colonisation competence of core gut microbiota in adulthood (Shi et al. 2025), revealing the long-term detrimental effects of antibiotics on honeybee health. The threat posed by potential ARGs demands considerable attention, as existing research demonstrates their greater abundance and diversity compared to known ARGs across all studied environments, forming a diversified reservoir from which new resistance determinants can be recruited by pathogens (Inda-Diaz et al. 2023), with the honeybee gut representing a significant aggregation site for such potential ARGs.

The core mechanism by which the honeybee gut serves as a hub for the dissemination of ARGs lies in its highly efficient horizontal gene transfer capability. Current studies have identified the sulfonamide resistance gene *sul2* in the honeybee gut, which exhibits high sequence homology to IncQ plasmids (Allara and Girard 2024). Furthermore, *sul2, strA*, and *strB* genes have been co-localised in contigs from both *Gilliamella* and *Snodgrassella* strains. These contigs also contain the origin of replication, mobilisation genes, and replication genes, forming genomic regions highly homologous to the IncQ plasmid RSF_1010_ (Sun et al. 2022). The IncQ plasmid family is renowned for its unique strand-displacement replication mechanism and its ability to function across a broad range of bacterial hosts (Loftie-Eaton and Rawlings 2012). *In vitro* conjugation experiments have confirmed that resistance genes such as *sul2* can be transferred among honeybee gut bacteria via mobilisable plasmids. These plasmids undergo distinctive evolutionary adaptations within the gut environment—characterised by large-fragment deletions in the *mobABC* and *repABC* regions, resulting in a “satellite plasmid” structure that paradoxically enhances their dissemination efficiency within the microbial community (Sun et al. 2022). Currently, our understanding of the evolutionary advantages of IncQ plasmids in the bee gut primarily stems from *in vitro* experiments and short-term observations. The long-term evolutionary path of these plasmids in authentic bee colony settings, along with whether their “satellite structures” will undergo convergent evolution in other bacterial hosts, remains an unresolved issue.

### Ecological risks of ARG dissemination in honeybees

Plasmid-mediated horizontal gene transfer establishes the honeybee gut as a reservoir of ARGs, posing threats to ecology and health through various pathways. Antibiotics, which are drugs used to treat or prevent bacterial infections, exert selective pressure that drives the accumulation and spread of these resistance determinants. First, in terms of colony health risks, resistance genes may transfer to bacterial pathogens such as *Paenibacillus larvae*, rendering conventional antibiotic treatments ineffective and potentially exacerbating colony collapse disorder (CCD; Vanengelsdorp et al. 2009). Elevated abundances of Gamma-proteobacteria have been observed in the gut microbial communities of bees from colonies affected by CCD (Dillon et al. 2010). The increase in Gamma-proteobacteria may play a beneficial role in enhancing the insect host’s resistance to gastrointestinal threats (Dillon et al. 2010). Second, regarding environmental dissemination pathways, bees can carry resistant bacteria into nectar during foraging activities. These drugs are prevalent in human-influenced settings such as agricultural lands and wastewater, where foraging bees are likely exposed. This integrate ARGs into plant–insect–soil cycles that amplify the resistance gene pool across ecosystems (Hassell et al. 2019). Previous studies have confirmed that NDM-β-lactamase-producing bacteria from poultry farms can spread to wild birds (Wang et al. 2017a). ESBL-producing *E. coli* has been detected in wild gulls feeding on human waste (Bonnedahl et al. 2009). Clinically relevant resistance genes have also been found in migratory birds with minimal human contact, facilitating their long-distance geographical spread (Cao et al. 2020). Thus, animals not only reflect the presence of resistance genes in contaminated environments but may also act as potential reservoirs and vectors for multidrug-resistant microbes (Radhouani et al. 2014). Finally, transmission through the food chain represents another risk; the abundance of ARGs in hive products shows a positive correlation with human-influenced environments, indicating that these genes likely originate from bee foraging habitats (Laconi et al. 2022). Consequently, honey and other hive products may become potential exposure routes for humans to antimicrobial-resistant bacteria.

It is noteworthy that the social structure of honeybees significantly accelerates the dissemination of ARGs within colonies. Worker bees carrying resistance plasmids can transfer resistant bacteria to nestmates through trophallaxis (Wang et al. 2024), rapidly establishing the entire colony as a “mobile reservoir” of resistance genes (Ludvigsen et al. 2018). This population-level transmission dynamic offers a natural model for studying the macroscopic spread of AMR. Future research utilising the honeybee model system will help explore mobile element-driven resistance transmission in gut environments and elucidate the *in vivo* evolutionary mechanisms of plasmid-mediated antibiotic resistance, thereby mitigating adaptive costs and favoring persistence and reproduction. The social transmission of ARGs through trophallaxis in bee colonies presents compelling parallels with the nosocomial spread of resistant pathogens in human healthcare settings (van Schaik 2015). Both systems involve high-density living, frequent contact, and shared “environments” (the hive or the hospital ward), suggesting that the honeybee model can generate broadly applicable insights into the ecology of resistance dissemination in structured populations.

## Environmental multidimensional interactions and colony health regulatory networks

This section integrates multidimensional environmental factors such as geography, climate, and aquaculture practices to construct an “environment microbe host health” interaction framework, revealing the specific pathways through which environmental pressure affects bee colony health through microbial communities.

### Geographical and climatic dimensions

Studies conducted across an altitudinal gradient of 200–1400 m in the Indian Peninsula revealed a U-shaped pattern in the gut microbiota alpha diversity of Eastern honeybees, with the lowest diversity observed at mid-altitude (600 m) and a unique microbial assemblage—including cold-adapted strains such as *Pantoea dispersa* and *A. kunkeei*—emerging at high altitudes (>1400 m) (Kwong and Moran 2016). This distribution pattern closely correlates with temperature gradients, suggesting that climate warming may reshape the altitudinal adaptation landscape of gut communities. Further evidence from seasonal variation shows that winter is associated with a marked shift in gut composition, where non-core bacteria become dominant and core gut bacteria are significantly reduced, a trend consistent with the increased abundance of *Bacillus* in plains regions as temperatures drop (Li et al. 2022). Investigating these bacterial networks helps elucidate honeybee disease mechanisms and host–microbe interactions, thereby supporting improved bee health and facilitating the discovery of novel molecules and enzymes with biotechnological potential (Romero et al. 2019). Recent research indicates that cold adaptation in honeybees critically depends on their gut microbiota. Cold-tolerant colonies exhibit enhanced lipid synthesis capabilities and more efficient acquisition of glucose and pyruvate, while actively supplying glucuronic acid and ascorbate to *Gilliamella* as carbon sources. In return, *Gilliamella* hydrolyzes pollen β-glucans into glucose and converts glucuronic and galacturonic acids into pyruvate, thereby providing essential energy substrates to the host. Interestingly, *Gilliamella* maintains restricted growth while degrading glucuronic acid and ascorbate, reflecting a finely tuned bidirectional resource-regulation mechanism (Tang et al. 2025). This discovery reveals how honeybees in cold regions leverage gut microbiota to regulate environmental adaptability, highlighting a sophisticated metabolic synergy between host and symbiont. Furthermore, regional antibiotic exposure also influences bee microbiota. A survey across 14 provinces in China showed that the abundance of ARGs in honeybees from the North China Plain agricultural zone was significantly higher than that in the Qinghai–Tibet Plateau region, correlating positively with local veterinary antibiotic usage intensity (Sun et al. 2022). Due to their limited foraging range and regional fidelity, *A. cerana* can serve as sensitive bio-indicators for monitoring local environmental AMR.

### Beekeeping practice dimensions

Divergent beekeeping practices significantly influence the honeybee gut resistome, where the migratory management of Western honeybees exposes them to diverse environmental resistance sources, resulting in a higher transferable abundance of ARGs compared to semi-wild managed Eastern honeybees (Sun et al. 2022). Regarding nutritional interventions, although artificial sugar supplementation does not markedly alter gut microbiota diversity in wild bumblebees, the addition of prebiotics such as inulin to commercial bee diets can promote the proliferation of *Bifidobacterium*, enhancing young bees’ resistance to microsporidian infection (Baffoni et al. 2016). Furthermore, the gut symbiont *Bombella* intestini, which constitutes nearly a quarter of the queen’s gut microbiota but is minimal in workers, extends worker lifespan by elevating tryptophan levels in larval food and activating the tryptophan-kynurenine pathway (Jin et al. 2025). Advancements in hive management also demonstrate beneficial effects: antibiotic-free hive treatments significantly increase the abundance of functional *Lactobacillus* strains in the queen’s gut, which is correlated with substantially improved queen survival rates (Kim et al. 2024).

### Pesticide and toxic plant stressors

Antibiotics such as oxytetracycline, tylosin, and fumagillin have been widely used in beekeeping across multiple countries and regions (Piva et al. 2020). Accumulating evidence indicates that antibiotic exposure adversely affects honeybee health by increasing susceptibility to pathogens and mortality, while impairing social behaviors (Raymann et al. 2018b, Daisley et al. 2020a, Raymann et al. 2017). Specifically, antibiotic treatment from larval to adult stages delays the behavioral development of foragers (Ortiz-Alvarado et al. 2020), which may be associated with alterations in lipid metabolism and neuroendocrine activity during both immature and adult stages (Ortiz-Alvarado et al. 2020, Ortiz-Alvarado and Giray 2022). Concurrently, antibiotics disrupt the gut microbial community (Raymann et al. 2018a) and induce immune deficiency in adult worker bees (Daisley et al. 2020a,b). Even subtherapeutic concentrations of tetracycline inhibit the growth of *Snodgrassella* in the honeybee gut, compromise the biofilm barrier, and increase the risk of pathogenic colonisation (Decourtye et al. 2005, Raine 2018, Stuligross and Williams 2020, de Castro Lippi et al. 2024). Regarding pesticide exposure, high-dose insecticide applications have been demonstrated to cause acute and widespread mortality in bees (Sanchez-Bayo and Goka 2014), while even sublethal doses lead to extensive adverse effects, including impaired foraging behavior, reduced cognitive ability, decreased weight gain, and lower reproductive success (Decourtye et al. 2005, Raine 2018, Stuligross and Williams 2020, de Castro Lippi et al. 2024). Neonicotinoid pesticides (e.g. clothianidin) induce oxidative stress in the honeybee gut, resulting in decreased abundance of *Gilliamella* and weakened polysaccharide metabolic function (Sacks et al. 2018). Neonicotinoids also impair the reproductive health of queens and drones, leading to reduced queen egg-laying capacity, decreased semen quality, and increased sperm mortality in drones (Wu-Smart and Spivak 2016). These neonicotinoid insecticides can interact with other stressors such as pathogens and parasites, exacerbating bee mortality and contributing to sharp declines in colony strength (Pettis et al. 2012, Di Prisco et al. 2013, Kang et al. 2024). For immature bees, exposure to sublethal concentrations of neonicotinoid pesticides reduces resistance levels and increases susceptibility to *Nosema* infection (Vidau et al. 2011). Pesticide exposure disrupts the composition of the gut microbiota, typically causing dysbiosis, most commonly characterised by reduced abundance of core microbiome members (Rouze et al. 2019, Motta et al. 2020, Zhu et al. 2020). Studies show that oral exposure to pesticides such as nitenpyram, coumaphos, glyphosate, and imidacloprid reduces the abundance of core bacterial genera, including *Gilliamella* and *Lactobacillus* (Rouze et al. 2019, Motta et al. 2020, Zhu et al. 2020), and their reduction significantly impacts honeybee gut health. For instance, *Gilliamella* plays crucial roles in digesting and metabolising various plant-derived carbohydrates and detoxifying food components (Raymann et al. 2018c, Engel et al. 2012), and it can enhance host health by priming the immune system against future pathogenic infections (Cariveau et al. 2014, Kwong et al. 2017). Additionally, *Lactobacillus* converts tryptophan into indole derivatives that activate the host aryl hydrocarbon receptor, thereby supporting memory formation (Zhang et al. 2022a). The reduction of these bacteria exerts notable negative effects on the honeybee host. Furthermore, toxic plants such as *Ziziphus Mill* (Ma et al. 2020), *Tilia cordata Mill*., and *Gastrodia* produce nectar that can directly cause acute poisoning or chronic toxicity in bees, leading to colony declines (Tang et al. 2023). Nectar from *Bidens pilosa* reduces *Bartonella* abundance in bees, while inoculation with *Bartonella apis* significantly enhances bee resistance to *B. pilosa* and upregulates immune-related genes. This indicates that the bee detoxification system possesses some resistance to toxic nectar plants like *B. pilosa*, and gut bacteria *B. apis* and *A. kunkeei* may assist in confronting such plant stress by enhancing host immunity (Tang et al. 2023).

**Figure 5:**
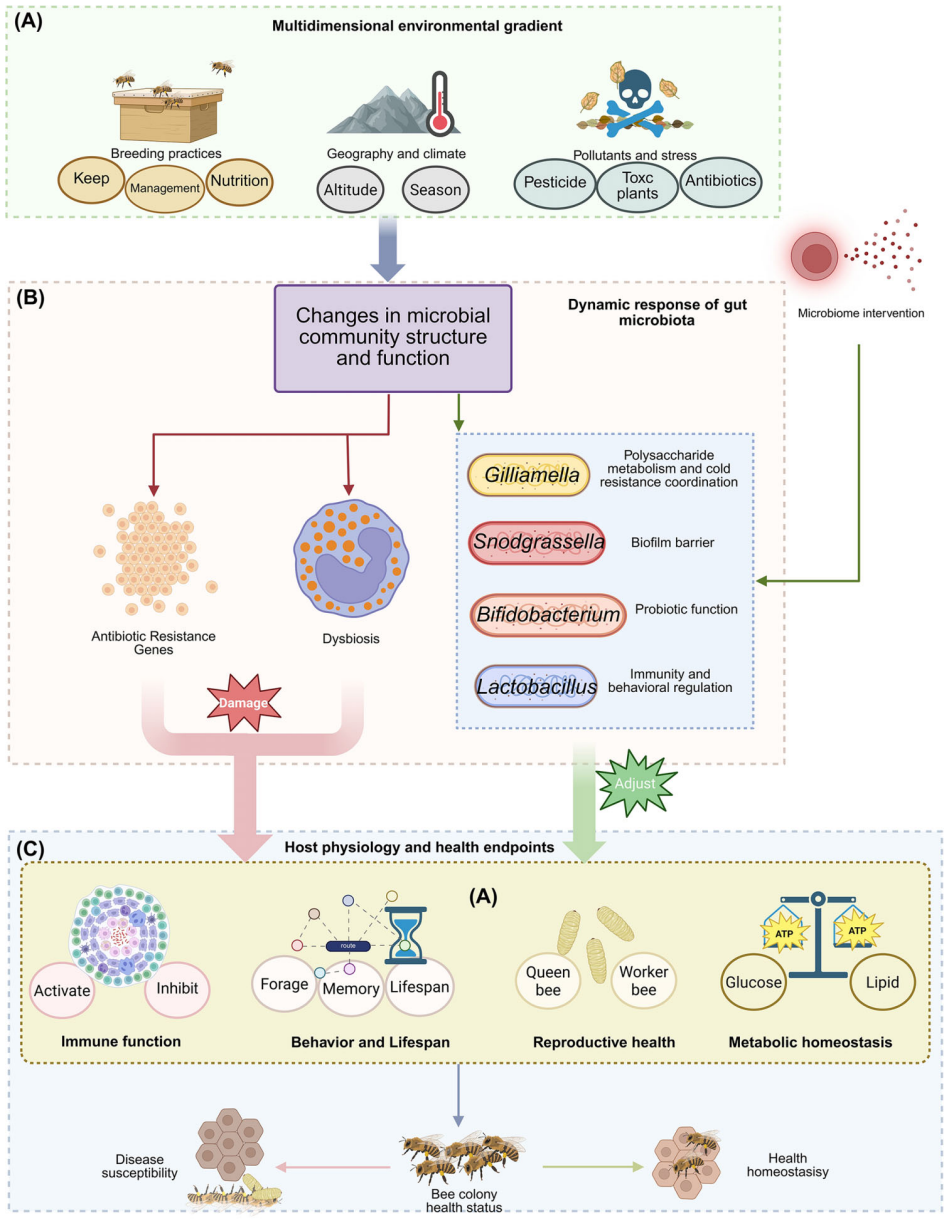
Response of bee gut microbiota under multidimensional environmental stress and its mechanism of impact on host health. (A) Multidimensional environmental gradient. This encompasses geographical and climatic factors such as altitude and season, as well as aspects of farming practices, including rearing, nutrition, management, and pollutants and stressors like antibiotics and toxic plants. (B) Dynamic response of gut microbiota. When stress from the environment enters the bee gut, it can, on one hand, lead to the enrichment of ARGs and dysbiosis; on the other hand, through microbiome intervention, bee gut microorganisms can regulate the gut microbiota structure and alleviate the disorder. (C) Host physiology and health endpoints. Changes in both microbial communities can have an impact on the host’s immune function, behavioral lifespan, and metabolic homeostasis, leading to the health or decline of bee colonies. Created with BioRender.

The honeybee gut microbiota is not a closed system; multidimensional environmental gradients dynamically shape its structure and function. This interaction reflects both the microbiota’s response to environmental changes and its regulatory role in host adaptation, collectively forming a complex health-impact network. These intricate health networks interact synergistically to maintain the coordinated development of the host–microbiota–environment triad. The effects of these multidimensional interactions ultimately converge on colony health outcomes. When environmental pressures exceed the buffering capacity of the microbiota, they can trigger a cascade of “microbiota dysbiosis–immune dysregulation–increased pathogen susceptibility.” As summarized in Fig. 5, the specific pathways through which environmental stressors affect host health via gut microbiota are illustrated. Consequently, understanding the environment–microbiota interaction network is fundamental to achieving precise management of honeybee colony health.

## Translational applications and prospective research directions of honeybee gut microbiota

The final chapter provides a systematic outlook on the transformation and application path from probiotic development, engineering bacterial technology to the global drug resistance monitoring network, providing innovative solutions for using bee models to solve practical problems.

### Colony health enhancement strategies through precision microbiota management

The development of probiotic formulations is advancing toward precision and region-specific customisation. For instance, utilising altitude-adapted strains such as highland-type A. *kunkeei* enables the design of “region-specific probiotics” tailored to support honeybee populations in distinct geographical areas. *Bifidobacterium*-based probiotics have been shown to significantly enhance the tolerance of Eastern honeybees to neonicotinoid pesticides, with field trials demonstrating markedly improved colony survival rates (Zhang et al. 2022b). Supplementation with honeybee-derived *Lactobacillus melliventris* significantly increases the survival and flower-visiting efficiency of *Bombus terrestris*, mediated by the upregulation of tyrosine metabolism and enhanced immune function (Zhang et al. 2022b). Moreover, probiotic mixtures containing core gut species can replenish the disturbed gut microbiota of worker bees, protecting them from *P. larvae* and reducing susceptibility to *Serratia marcescens* (Powell et al. 2021). The application of *Lactobacillus johnsonii* in honeybees—through metabolites produced by the probiotic—increases hive population and fat body reserves, thereby better controlling the proliferation of *N. ceranae* (Maggi et al. 2013). In the field of engineered bacterial anti-pathogen technology, genetic modification of *S. alvi* has yielded a system capable of inducing RNA interference (RNAi) within the host. Through dsRNA expression, the engineered strain effectively silences genes of both *Varroa destructor* and Deformed wing virus (DWV), thereby suppressing infection by DWV and mites (Lariviere et al. 2023). Further research has revealed that *S. alvi* can inhibit *N. ceranae* proliferation by stimulating the host’s oxidant-mediated immune response. During infection, *N. ceranae* relies on the thioredoxin and glutathione systems to counteract oxidative stress and maintain redox balance. Nanoparticle-mediated RNAi targeting γ-glutamylcysteine synthetase and thioredoxin reductase genes of the parasite significantly reduces spore load, confirming the critical role of antioxidant mechanisms in *N. ceranae* invasion. By engineering the symbiotic bacterium *S. alvi* to deliver dsRNA targeting genes involved in the spore redox system, RNAi is induced, parasite gene expression is suppressed, and microsporidian parasitism in bees is significantly inhibited (Lang et al. 2023b). The deployment of engineered *S. alvi* for RNAi-based pathogen control, while promising, necessitates rigorous pre-release risk assessment. Key considerations include the horizontal gene transfer potential of the engineered constructs to native microbiota and the long-term fitness impact on host colonies—issues that must be addressed through contained field trials and modeling before broader application. In the field of disease-resistant honeybee breeding, studies have confirmed that *A. cerana* strains selected based on gut microbiota composition exhibit stronger resistance to *N. ceranae* infection. This provides a novel strategy for disease-resistant breeding, where characteristics of the core gut microbiota—such as *Lactobacillus* abundance and *Gilliamella* diversity—can be incorporated as selection criteria. Combined with modern genomic technologies, this approach enables the cultivation of honeybee strains with enhanced resistance (Kaskinova et al. 2023, Yang et al. 2025). Furthermore, *A. kunkeei* and *Bifidobacterium* sp. isolated from the gut of Thai honeybees were shown to significantly reduce mortality, spore load, and infection rate in giant *Apis dorsata* challenged with *N. ceranae*, while also increasing hypopharyngeal gland protein levels (Naree et al. 2025). These findings provide a new direction for the biological control of honeybee diseases as an alternative to antibiotics, offering new avenues for antibiotic-free disease management and resistant stock breeding.

Therefore, although microbial intervention strategies represented by probiotic formulations, engineered bacteria technology, and disease-resistant breeding show great promise for precise health management, their path to widespread application still faces a series of bottlenecks that need to be urgently broken through (Pradeep et al. 2025). First, the environmental stability and long-term efficacy of the technology remain in question: whether the introduced non-native strains or engineered bacteria can stably colonise and maintain their functions in the complex and variable wild bee colonies, and whether there is a risk of horizontal gene transfer of their genetic elements to the local microbiota, all need to be evaluated through long-term field monitoring (Zhao et al. 2025). Secondly, ecological safety and long-term impacts are core concerns, including the non-target effects of engineered organisms, the potential cost to the overall adaptability of bee colonies, and the strict regulatory and public acceptance challenges faced by their release. Finally, there are significant knowledge gaps in current research regarding the mechanism of action and personalised application, such as the precise interaction mechanism between probiotics and host physiology, personalised intervention plans based on host genotypes and native microbiota, and the lack of a standardised long-term evaluation system (Pradeep et al. 2025). Therefore, future translational research must, while actively developing applications, be committed to transforming these challenges into a roadmap for the field to mature and become reliable through rigorous field trials, in-depth mechanism analysis, and comprehensive risk assessment.

### Novel antimicrobial molecule discovery and resistance control

The construction of antimicrobial peptide libraries has enabled the isolation of novel AMPs such as Apilactocin-1 from secondary metabolites of honeybee gut symbionts like *Lactobacillus apis*, demonstrating superior efficacy against multidrug-resistant *P. larvae* compared to conventional antibiotics. Genomic analysis of 477 honeybee gut bacterial genomes identified over 700 novel secondary metabolite BGCs, with the core symbiont *Gilliamella apis* producing ribosomally synthesised and post-translationally modified peptides (RiPPs) that significantly inhibit *Melissococcus plutonius* growth—the 10-amino-acid core peptide exhibiting exceptionally high antibacterial activity against Gram-positive bacteria, establishing a foundation for developing microbiota-derived natural products as biocontrol agents against bee diseases (Lang et al. 2023a). Concurrently, plasmid transmission blockers have been developed through antisense oligonucleotides (ASOs) targeting the *repABC* replication initiation region, inhibiting plasmid replicase activity and reducing IncQ plasmid conjugation efficiency in the honeybee gut (Yang et al. 2022). IncQ plasmid replication is coordinately regulated by *repA, repB*, and *repC* proteins, with *repC* specifically recognising the origin of replication (*oriV*). ASOs designed to target the 5’-UTR region of *repC* mRNA (e.g. sequence 5’-AUGACUGC-3’) sterically hinder ribosome binding and prevent replication initiation complex assembly (Sun et al. 2022). Furthermore, environmental resistance monitoring networks are being established through geographic information systems (ARGs-GIS) based on the gut resistome of *A. cerana* to real-time monitor regional antibiotic contamination hotspots. The innovation of this ARGs-GIS platform lies in integrating biological indicators (microbial resistome) (Li et al. 2025), environmental correlates (pesticides/heavy metals) (Ju et al. 2023), and spatial information technologies, achieving a paradigm shift from passive monitoring to proactive prevention of antibiotic pollution. Future development requires cross-species integration, AI-enabled analytics, and blockchain-based verification to advance this system into key infrastructure for containing resistance dissemination within the “One Health” framework.

### Cross-species health research models

The simplified ecosystem of the honeybee gut provides a unique reference model for human intestinal research. Its strain-level immune recognition mechanisms, such as the “dual-pathway recognition–precision ROS clearance” model, offer novel insights into understanding the selective secretion of IgA toward commensal bacteria in the human gut. Human TLR4 activates IgA secretion in B cells via MyD88 (Wang et al. 2017b), whereas the honeybee Toll pathway homolog *dorsal-1* may regulate ROS production (Guo et al. 2023), indicating conserved pathway architecture across species. Clinical studies have revealed that elevated DUOX2 expression in the gut of ulcerative colitis patients coincides with disrupted IgA secretion, suggesting potential crosstalk between ROS and IgA regulation (Guo et al. 2023). In the context of neuro-microbial interactions, under stress from the pesticide nitenpyram, *Bifidobacterium* activates intestinal gluconeogenesis through microbial metabolites, promoting succinate production, which subsequently stimulates brain ILP1 expression and repairs pesticide-induced oxidative metabolic damage. This process depends on a “succinate–intestinal gluconeogenesis-brain ILP1” axis that is homologous to mammalian insulin regulatory mechanisms (Han et al. 2024). These findings demonstrate that metabolites from honeybee gut *Bifidobacterium* influence neuropeptide expression, providing a simplified model for studying the “insect gut–brain axis.” Furthermore, the strain *Gilliamella* sp. G0441 enhances host pesticide tolerance via the microbiome–gut–brain axis (MGBA) by upregulating intestinal esterase E4 gene expression, activating cerebral lipid metabolism pathways, and restoring ascorbate metabolism suppressed by nitenpyram (Zhao et al. 2025). These results underscore the significant role of *Gilliamella* in honeybee resilience to neonicotinoids and provide insights into the MGBA as a pathway for enhancing pesticide tolerance and ecological health. Moreover, allotransplantation of the queen-associated bacterium *Bombella* intestini into worker bees extends lifespan and enhances immunity through a “microbe-metabolite-host” axis (Jin et al. 2025). Research has confirmed that the invasive *E. coli* strain LF82, which is associated with human Crohn’s disease, can induce increased intestinal permeability, tryptophan metabolism disorders, and significant impairment of learning and memory abilities in bees after infection. This complete pathway of “intestinal inflammation—metabolic changes—cognitive impairment” is highly similar to the cognitive decline often accompanying IBD patients, thereby establishing bees as a novel experimental model for studying the interaction mechanism of the IBD-related gut–brain axis (Chang et al. 2022). At the same time, the bee model has also demonstrated universal principles in the study of metabolic diseases: it was found that the core intestinal bacterium *Lactobacillus Firm-5* ferments to produce succinic acid, activates intestinal gluconeogenesis, and subsequently upregulates the expression of insulin-like peptides in the brain to maintain host blood glucose homeostasis; this “microbial metabolite—intestine—brain” regulatory axis shares core commonalities with the insulin secretion regulation in mammals, providing a brand-new mechanistic perspective for the exploration of microbial intervention strategies for human diabetes and other metabolic diseases (Han et al. 2024). These specific cases indicate that the study of bee intestinal microbiota goes beyond the realm of insect biology, and the fundamental biological principles it reveals—including the systemic regulation of host immunity, metabolism, and neural function by microorganisms—can be directly analogised and inspire research on the pathological mechanisms and potential therapeutic targets of human chronic inflammatory diseases and metabolic disorders.

### Comparative perspectives: insect symbiosis models

While the honeybee gut microbiome provides a premier model for social transmission and host–microbe dialogue, placing its insights within the broader spectrum of insect symbiosis reveals both unique and convergent evolutionary solutions. Examining other well-studied systems, such as aphids, whiteflies, and ants, enriches the proposed “Environment-Microbiota-Host” framework and underscores the potential for cross-system synthetic biology applications (Navarro-Escalante et al. 2025). Aphids exemplify an extreme of obligate intracellular symbiosis, dominated by the vertically transmitted, genomically reduced Buchnera. Notably, aphids also harbor horizontally acquired facultative symbiont, which can rapidly confer context-dependent benefits like heat tolerance or parasite resistance (Luttenschlager et al. 2025). This highlights a layer of ecological plasticity distinct from the bee’s stable core microbiome and illustrates how facultative partners can mediate complex multi-trophic interactions (Luttenschlager et al. 2025). Building on the theme of intracellular complexity, whiteflies present a remarkable case of multi-partner consortia within a single host cell. Crucially, these symbiotic systems are dynamic and subject to genetic innovation, as evidenced by the finding that horizontal gene transfer events (e.g. of plant-derived thaumatin-like protein genes) can drive ecological niche differentiation in closely related whitefly species (Hu et al. 2025). This mirrors the honeybee gut resistome’s role as an environmental sensor and affirms a universal principle of microbiome and symbiotic gene repertoire-mediated environmental interaction. Transitioning to another eusocial insect, ants demonstrate how symbiosis underpins dietary specialisation and ecological success. Through associations with gut microbes and endosymbiont, ants achieve nitrogen recycling and plant polymer degradation (Hu and Moreau 2025). The maintenance of these communities is facilitated by intensive social interactions, offering a powerful parallel to bees in demonstrating sociality as an engine for microbial homeostasis, while showcasing divergent nutritional alliances that enabled niche expansion (Hu and Moreau 2025). Collectively, these comparative systems illustrate a continuum of symbiotic strategies. They affirm that host ecology, diet, and social structure are fundamental architects of microbial partnerships, thereby contextualising the honeybee model within a wider evolutionary narrative. Importantly, the principles learned from these diverse systems are now converging in the field of engineered symbiosis, where synthetic microbial communities (SynComs) are being rationally designed for sustainable insect management (Ye et al. 2025), broadening the potential for microbiome-based interventions across insect systems.

## Conclusion and future perspectives

Research on the honeybee gut microbiota has undergone a profound transformation, facilitated by the system’s inherent experimental accessibility and genetic tractability (Sun et al. 2022). These attributes have propelled the field from descriptive studies to the active construction of intervention technologies and the dissection of multidimensional interaction networks. Looking forward, the operational advantages of this model, such as suitability for real-time tracing of gene transfer and deployment in multi-trophic resistance interruption strategies, will be instrumental in transitioning from observation to the development of integrated One Health solutions. For example, engineered *Snodgrassella* expressing dsRNA targeting microsporidia can reduce spore parasitism (Sun et al. 2022), and CRISPR-dCas9 labeling systems enable real-time tracking of plasmid transfer dynamics in the ileal biofilm region (Lamarthee et al. 2023), transforming honeybees from “observation subjects” into “mechanistic validation platforms.” More importantly, the field is transitioning from basic exploration to integrated One Health solutions, with research objectives directly addressing resistance control, colony health, and ecological security. Probiotic *Gilliamella* sp. G0441 enhances colony survival via a gut–brain axis mechanism involving upregulation of esterase E4 for pesticide degradation, repair of neuro-metabolic damage, and suppression of the Mitogen-Activated Protein Kinase inflammatory pathway (Zhao et al. 2025). Multitrophic intervention strategies and antisense oligonucleotides that inhibit plasmid replication help build a closed-loop intervention system spanning “environment-insect-soil.” Furthermore, the ARGs-GIS monitoring network based on *A. cerana* enables precise mapping of global contamination hotspots and supports targeted governance responses.

However, current applications based on the honeybee model organism remain insufficiently widespread. Future efforts should focus on actively integrating existing research technologies to achieve comprehensive visualisation of the host–microbiota–environment triad, aiming for breakthroughs in understanding coevolution and predicting ecological responses to environmental changes. Specifically, advancement can be pursued through three key approaches: Recently, real-time tracing techniques for horizontal gene transfer can be established to verify the feasibility of the hypothesis. A plasmid dynamic labeling system based on CRISPR-dCas9 can be developed to visualise the spread of ARGs in live bees. The CRISPR-dCas9 system (Yang et al. 2018), through inactivation of Cas9 nuclease (D10A/H840A mutations) while retaining DNA-binding capacity, functions as a “genetic locator.” For example, embedding MS2/PP7 RNA aptamers into the sgRNA stem-loop structure enables recruitment of fluorescent protein-fused capsid proteins (e.g. GFP/mCherry), achieving dual-channel fluorescence labeling of both plasmid DNA and recipient bacteria (Wang et al. 2016). This labeling system facilitates visualisation of horizontal gene transfer, significantly enhancing observation and investigation of resistance gene transmission. Research on the difficulties of blocking multi-trophic-level drug resistance transmission in the middle and later stages can be conducted. Design a “environment-plant-insect-soil” antibiotic resistance transmission blocking strategy based on bees to form an ecological closed-loop control. Implementation involves a quadruple intervention approach comprising source reduction (environment) (Guo and Zhang 2017, Jeon et al. 2023), intestinal intervention (insect) (Sun et al. 2022, Muntaabski et al. 2025), physical barriers (plant) (Jeon et al. 2023), and *in situ* inactivation (soil) (Munir et al. 2011, Xiang et al. 2019, Paruch et al. 2021), effectively disrupting the ecological cycle of resistance genes. Future development should further integrate smart monitoring (honeybee ARGs-GIS), synthetic biology tools (CRISPR-based anti-resistance plasmids), and circular agricultural models (composting-phytoremediation) to construct sustainable agricultural ecosystems with low resistance risks. Finally, a global bee gut microbiome program can be launched to conduct cross-disciplinary real-time monitoring in collaboration with geography, mathematics, and genomics. For instance, establishing a cross-continental bee intestinal sample bank and drug resistance gene database, and mapping out the global distribution and evolution of drug resistance. This requires multidimensional data integration encompassing *A. cerana, A. mellifera*, and *Bombus* spp. to analyze interspecies differences in resistomes, such as the lower carriage rate of *strA* genes in *A. cerana* compared to *A. mellifera*. Simultaneous documentation of pesticide usage intensity, heavy metal pollution indices, and veterinary antibiotic sales at sampling sites will enable the construction of “environmental pressure-microbiota resistance” response models (Sun et al. 2022). Research on the honeybee gut microbiome has transcended the scope of pollinator health, emerging as a strategic frontier for addressing the antibiotic resistance crisis, elucidating host–microbe coevolution, and predicting ecological responses to environmental changes. This review establishes the honeybee gut not merely as a model for symbiosis studies, but as a pioneering system that decodes universal rules governing AMR flow in ecosystems. By leveraging this system, we can transition from reactive monitoring to proactive containment of environmental AMR, thereby safeguarding both pollinator health and global food security—a critical step toward achieving the One Health ideal.
